# The TLR-4 agonist adjuvant, GLA-SE, improves magnitude and quality of immune responses elicited by the ID93 tuberculosis vaccine: first-in-human trial

**DOI:** 10.1038/s41541-018-0057-5

**Published:** 2018-09-04

**Authors:** Rhea N. Coler, Tracey A. Day, Ruth Ellis, Franco M. Piazza, Anna Marie Beckmann, Julie Vergara, Tom Rolf, Lenette Lu, Galit Alter, David Hokey, Lakshmi Jayashankar, Robert Walker, Margaret Ann Snowden, Tom Evans, Ann Ginsberg, Steven G. Reed, The TBVPX-113 Study Team, Jill Ashman, Zachary K. Sagawa, D. Tait, Sadritdin Ishmukhamedov, Gretta Blatner, Sharon Sutton, Barbara Shepherd, Casey Johnson

**Affiliations:** 1 Infectious Disease Research Institute, Seattle, WA 98102 USA; 2 Department of Global Health, University of Washington, Seattle, WA 98195 USA; 3 PAI Life Sciences, Seattle, WA 98102 USA; 4 Aeras, Rockville, MD USA; 5 Ragon Institute of Massachusetts General Hospital, Massachusetts Institute of Technology, Harvard University, Boston, MA 02139 USA; 6 Aeras, Cape Town, South Africa; 7 Johnson County, Clin-Trials, Lenexa, KS USA

## Abstract

Tuberculosis (TB) is the leading cause of infectious death worldwide. Development of improved TB vaccines that boost or replace BCG is a major global health goal. ID93 + GLA-SE is a fusion protein TB vaccine candidate combined with the Toll-like Receptor 4 agonist adjuvant, GLA-SE. We conducted a phase 1, randomized, double-blind, dose-escalation clinical trial to evaluate two dose levels of the ID93 antigen, administered intramuscularly alone or in combination with two dose levels of the GLA-SE adjuvant, in 60 BCG-naive, QuantiFERON-negative, healthy adults in the US (ClinicalTrials.gov identifier: NCT01599897). When administered as 3 injections, 28 days apart, all dose levels of ID93 alone and ID93 + GLA-SE demonstrated an acceptable safety profile. All regimens elicited vaccine-specific humoral and cellular responses. Compared with ID93 alone, vaccination with ID93 + GLA-SE elicited higher titers of ID93-specific antibodies, a preferential increase in IgG1 and IgG3 subclasses, and a multifaceted Fc-mediated effector function response. The addition of GLA-SE also enhanced the magnitude and polyfunctional cytokine profile of CD4^+^ T cells. The data demonstrate an acceptable safety profile and indicate that the GLA-SE adjuvant drives a functional humoral and T-helper 1 type cellular response.

## Tuberculosis: novel vaccine formulation elicits strong immune responses

A tuberculosis vaccine containing an immunity-potentiating agent stimulated strong immune responses in a first-in-human trial. Tuberculosis (TB) is the world’s foremost cause of infectious disease deaths, yet lacks an effective vaccine for adult humans. Rhea Coler, of the Infectious Disease Research Institute, Seattle, and a team from the United States and South Africa, tested their prophylactic on 60 healthy US adults. The vaccine consisted of ID93, a fusion of TB therapeutic target proteins, and GLA-SE—a supplement to boost immune responses. The candidate proved safe in all participants, with mild-to-moderate adverse effects, and provoked promising immune responses. The formulation was significantly more effective with GLA-SE than without. Further studies will elucidate the therapeutic benefit of this formulation and its ability to combat the pathogenicity of TB.

## Introduction

Tuberculosis (TB) continues to be the leading infectious disease killer, with 10.4 million new cases and 1.7 million deaths in 2016.^1^ Current treatment regimens are lengthy and challenging to complete; recurrences (relapse or re-infection) and drug resistance complicate an increasing number of cases. Progress fighting TB has stalled and new approaches to reducing the global TB burden are necessary. A vaccine against TB could serve to prevent primary infections, reduce the rate of progression to active TB, or augment chemotherapy to shorten treatment duration or increase treatment efficacy. The only licensed TB vaccine, Bacille Calmette-Guérin (BCG), is effective in preventing disseminated forms of TB in children but incompletely prevents infection or disease in adults.^2–4^ Development of improved TB vaccines that boost or replace BCG is a major global health goal.^1^

Human immune correlates of protection against TB have not yet been identified. T-helper 1 (Th1) type cellular immunity is known to be crucial for controlling *Mycobacterium tuberculosis* (Mtb) infection^5–10^ and thus vaccine strategies aim to elicit these subsets. Recently, studies have shown evidence that antibodies may also contribute to controlling disease in latently infected individuals.^11,12^

ID93 is a subunit TB vaccine candidate comprised of four antigens representing different families of Mtb proteins. Rv1813 is a conserved hypothetical protein that is upregulated under hypoxic growth and predicted to be localized in the outer membrane.^13^ Rv2608 (PPE42) is a probable outer membrane-associated PPE (Pro-Pro-Glu (PPE) motif-containing) protein.^14^ Rv3619 (EsxV) and Rv3620 (EsxW) are secreted proteins belonging to the ESAT-6 family of virulence factors.^15^ The four ID93 antigens have been shown to be recognized in Mtb-exposed individuals.^16,17^ ID93 is combined with the Th1-inducing synthetic TLR4-agonist adjuvant, Glucopyranosyl Lipid A (GLA), formulated in a stable oil-in-water nano-emulsion (SE).^18^ Prophylactic immunization with ID93 + GLA-SE has been shown to limit experimental infection of drug-sensitive and drug-resistant Mtb in mice and guinea pigs.^17,19–21^ Therapeutic immunization with ID93 + GLA-SE improved outcomes over antibiotics alone in mice and non-human primates.^22^ In this first-in-human, dose-finding, phase 1 clinical trial, we evaluated the safety and immunogenicity of ID93 + GLA-SE in a non-TB-exposed population.

## Results

### Subjects

Sixty volunteers were enrolled and randomized to receive three study injections. Fifty-six participants received all three injections and completed the Day 84 visit; 54 participants completed the final Day 420 visit. Of the six subjects who did not complete the study, none withdrew due to adverse events (AEs); five withdrew consent (four due to work schedule conflict, one due to relocation) and one was lost to follow-up.

### Safety

The vaccine was safe and well tolerated, with no SAEs or AEs of special interest considered related to treatment. The majority of subjects had mild or moderate AEs. One subject had a severe AE of transient, self-limited injection site erythema after the third injection of 2 μg ID93 + 2 μg GLA-SE. The erythema occurred 3 days after the third study injection and resolved by the next day. Between 33.3 and 100% of subjects reported at least one related AE in each treatment regimen (Table 1). The most common related AEs overall were injection site pain (76.7%), headache (28.3%), and fatigue (21.7%). Injection site pain was reported at a higher incidence in the ID93 + GLA-SE treatment regimens (ranging from 83.3 to 100%) compared to the ID93 alone regimens (33.3% for 2 μg; 16.7% for 10 μg). There was no apparent increase in frequency or severity of individual solicited or unsolicited AEs or AEs overall with successive or higher doses of ID93 antigen or GLA-SE adjuvant. These data indicate that the vaccine was safe and well tolerated at all dose levels tested.

**Table 1:** Safety evaluations—related adverse events

Finding	ID93					
	2 µg	2 µg	2 µg	10 µg	10 µg	10 µg
	GLA-SE					
	–	2 µg	5 µg	–	2 µg	5 µg
	*n* = 6	*n* = 12	*n* = 12	*n* = 6	*n* = 12	*n* = 12
Subjects with at least one related AE	3 (50%)	12 (100%)	10 (83%)	2 (33%)	12 (100%)	10 (83%)
Local solicited adverse events						
Injection site erythema	0	2 (17%)	0	0	0	0
Injection site pain	2 (33%)	11 (92%)	10 (83%)	1 (17%)	12 (100%)	10 (83%)
Injection site swelling	0	1 (8%)	1 (8%)	0	1 (8%)	0
Systemic solicited adverse events						
Arthralgia	0	0	1 (8%)	0	0	1 (8%)
Chills	0	2 (17%)	0	0	0	0
Decreased appetite	0	2 (17%)	1 (8%)	0	0	0
Fatigue	1 (17%)	4 (33%)	3 (25%)	0	1 (8%)	4 (33%)
Headache	2 (33%)	3 (25%)	3 (25%)	1 (17%)	2 (17%)	3 (25%)
Myalgia	0	2 (17%)	1 (8%)	0	0	2 (17%)
All other related AEs occurring in at least two subjects						
Aspartate aminotransferase increased	0	0	0	0	2 (17%)	0

### Vaccine antibody responses by ELISA

**Fig. 1:**
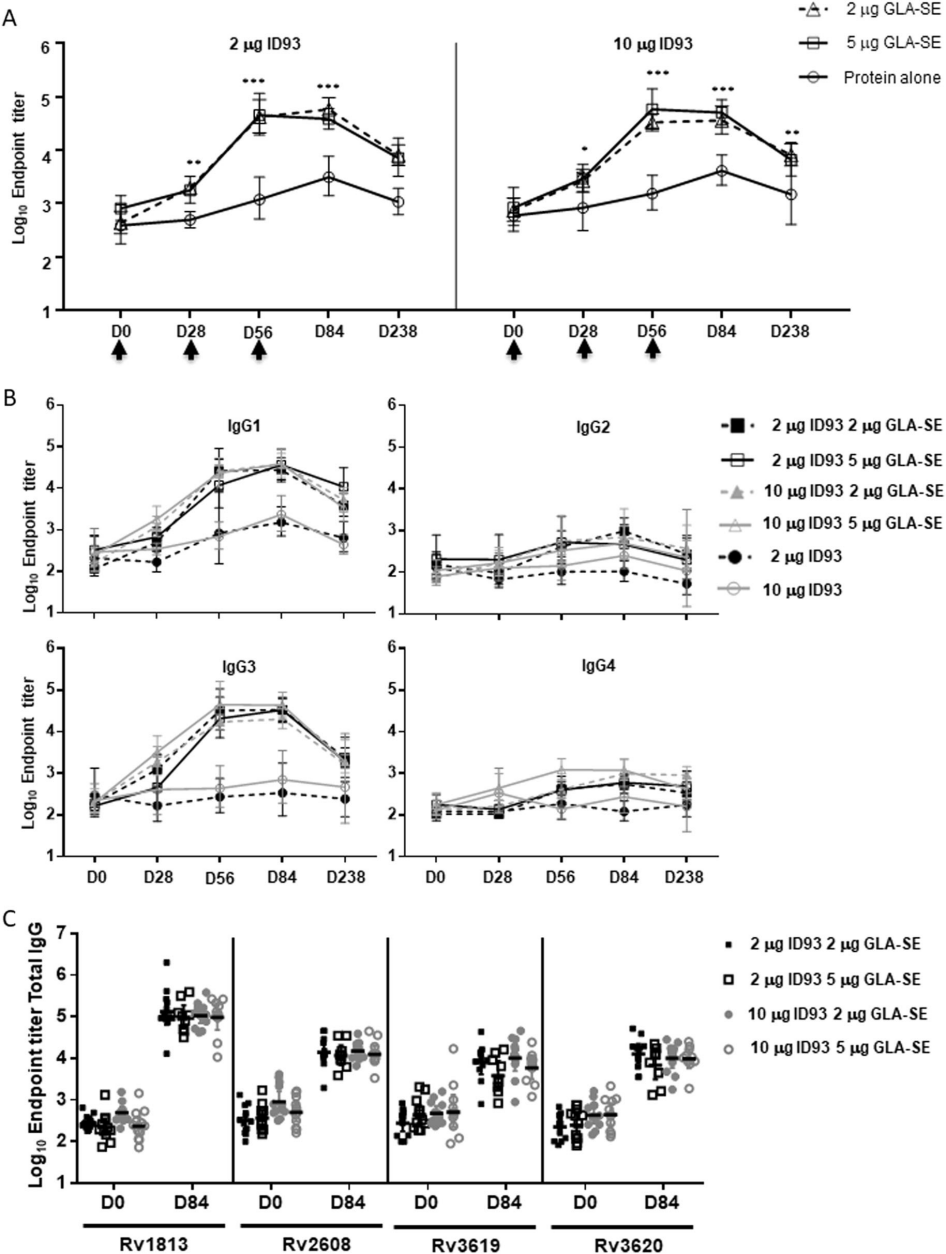
ID93 + GLA-SE elicited significantly higher ID93-specific total IgG antibody responses than those vaccinated with ID93 protein alone. **a** Magnitude of ID93-specific antibody responses for differing doses of antigen and/or adjuvant. Geometric mean titer and 95% confidence interval as determined by ELISA using serum collected on the days shown. Vaccinations were administered on Days 0, 28, and 56 (arrows) after blood collection. ID93 + GLA-SE elicited significantly higher ID93-specific total IgG antibody responses than those vaccinated with ID93 protein alone. Statistical significance between adjuvant containing regimens and protein alone regimens at each time point was evaluated by two-way ANOVA and Tukey’s multiple comparisons test with *p-*values indicated as: <0.05 (*), <0.01 (**), <0.0001 (***). **b** IgG subclass analysis of ID93-specific antibody responses for different antigen and adjuvant doses. Geometric mean titer and 95% confidence interval as determined by ELISA using serum collected at the days shown. Vaccinations were administered on Days 0, 28, and 56. Serum samples were collected on those days prior to study injections. Inclusion of the GLA-SE adjuvant elicited a preferential induction of IgG1 and IgG3 subclasses. **c** Component antigen specificity of antibody responses for individuals administered different antigen and adjuvant doses. Bar represents group geometric mean titer as determined by ELISA using serum collected at the days shown. Vaccinations were administered on Days 0, 28, and 56. Serum samples were collected on those days prior to study injections. Total IgG responses were measured against all four component antigens with Rv1813 being the highest

All ID93 + GLA-SE recipients had significantly higher vaccine-specific IgG titers compared to those receiving ID93 alone after a single vaccination and at every visit thereafter (Fig. 1a). Peak responses were observed after two vaccinations in the adjuvant-containing groups compared to three vaccinations for the ID93 alone groups. All antigen-adjuvant dose combinations induced similar IgG titers (Fig. 1a). ID93-specific total IgG responses persisted through Day 238 at >seven-fold over baseline in the GLA-SE groups compared to <three-fold in the ID93 alone groups. IgG subclass analysis revealed a predominance of IgG1 and IgG3 subtypes for the GLA-SE containing groups with no differences across different dose combinations (Fig. 1b). Antigen-specific IgG responses were induced against all four ID93 component antigens in all subjects receiving ID93 + GLA-SE. Median IgG titers against Rv1813 were the highest while the remaining antigens induced similar antibody levels (Fig. 1c).

### Vaccine-specific CD4^+^ T cell response profiles in stimulated PBMCs

**Fig. 2:**
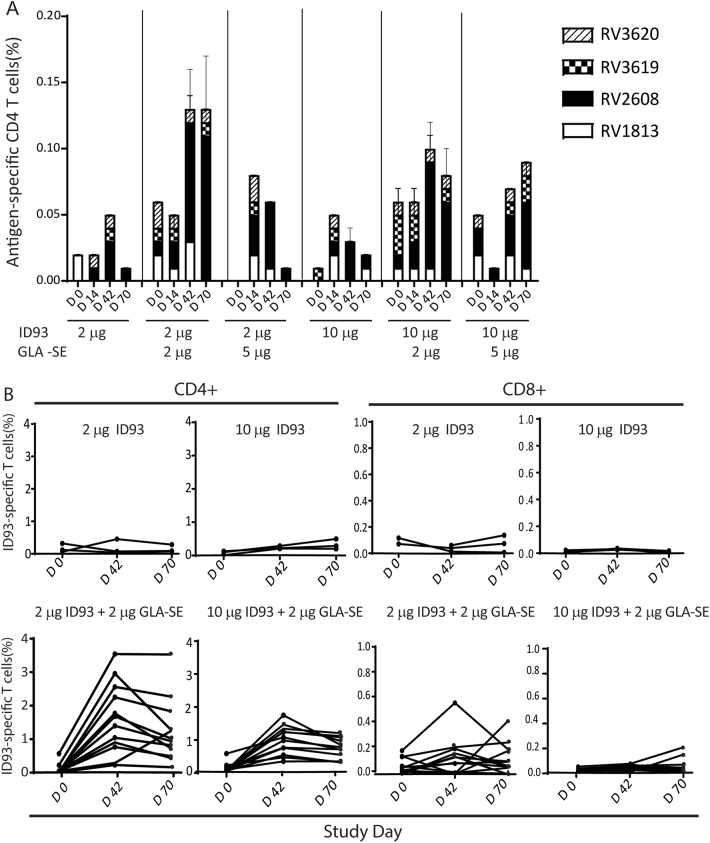
Magnitude and specificity of antigen-specific T cell response. **a** Kinetics of ID93 antigen-specific CD4^+^ T cells measured from cryopreserved PBMC at baseline and 2 weeks after each vaccination. Median frequencies of CD4^+^ T cells positive for any antigen-specific marker (IFNγ, TNF, IL-2, CD154, IL-4/IL-22, and/or IL-17) as measured by intracellular cytokine staining of antigen (peptide pools)-stimulated PBMCs with unstimulated values subtracted. Error bars show 95% confidence intervals. Vaccinations were administered on Days 0, 28, and 56. **b** Frequencies of ID93-specific (defined as positive for CD154, IFNγ, TNF, and/or IL-2) for CD4^+^ and CD8^+^ T cell responses in individual subjects as measured by intracellular cytokine staining of ID93 protein-stimulated whole blood with unstimulated values subtracted. Vaccinations were administered on Days 0, 28, and 56

Antigen-specific background-subtracted CD4^+^ T cell frequencies increased after vaccination in all groups with the highest responses observed in the adjuvant-containing groups (Fig. 2a). The majority of vaccine-specific CD4^+^ T cells were specific for Rv2608. The strongest two-week post-vaccination CD4^+^ T cell response was observed for the 2 μg ID93 + 2 μg GLA-SE regimen, with a peak median frequency after the second injection at Day 42 (0.11% in subjects who received all three study injections). The most prominent CD4^+^ T cell responses against Rv2608 were a mixture of polyfunctional (IL-2^+^TNF^+^CD154^+^), bifunctional (IL-2^+^CD154^+^ or TNF^+^CD154^+^), and monofunctional (IFNγ^+^ or CD154^+^ or IL-2^+^) responses. Minimal CD4^+^ T cell responses were detected against Rv1813, Rv3619, or Rv3620. CD8^+^ T cell responses against any of the four vaccine antigens were also minimal (data not shown).

### Vaccine-specific T cell response profiles in stimulated whole blood

**Fig. 3:**
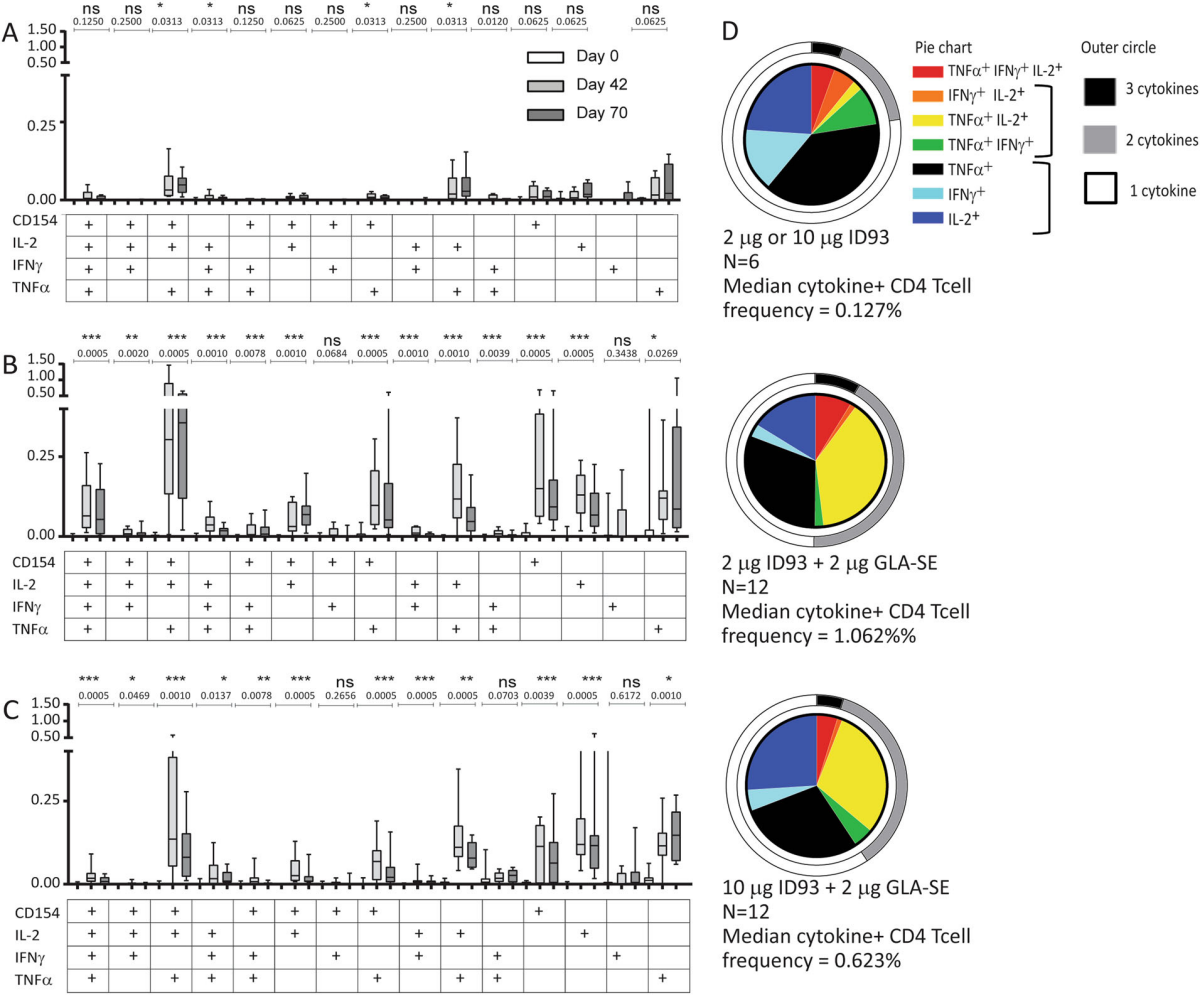
T cell cytokine profiles from whole blood ICS for Cohorts 1 and 2. Frequencies of ID93-specific CD4 T cells co-expressing different cytokines are shown for Cohorts 1 and 2 as measured by intracellular cytokine staining of ID93-stimulated whole blood with unstimulated values subtracted. The bar shows the median frequency, the box shows the interquartile range, and the whiskers show the maximum and minimum values. Vaccinations were given on Days 0, 28, and 56. **a** Combined data for 2 μg and 10 μg ID93 recipients, **b** 2 μg ID93 + 2 μg GLA-SE, **c** 10 μg ID93 + 2 μg GLA-SE. Wilcoxon matched pairs signed rank test to compare frequencies of ID93-specific cytokine response between Day 0 and Day 42 timepoints. **d** Proportions of ID93-specific CD4 T cells co-expressing different cytokines are shown for Cohort 1 and 2 subjects administered 2 μg ID93, 10 μg ID93, 2 μg ID93 + 2 μg GLA-SE, or 10 μg ID93 + 2 μg GLA-SE as measured by intracellular cytokine staining of ID93-stimulated whole blood with unstimulated values subtracted. Results are shown for Day 70 (2 weeks post third vaccination). Due to low subject numbers, results for 2 μg ID93 and 10 μg ID93 were combined

The highest CD4^+^ T cell frequencies were observed 14 days after the second vaccination in the adjuvant containing groups (Fig. 2a). Median peak CD4^+^ T cell responses were higher for the 2 μg ID93 + 2 μg GLA-SE group (1.54%) than the 10 μg ID93 + 2 μg GLA-SE group (0.99%), and minimal for the protein only groups. CD8^+^ T cell response were low for the 2 μg ID93 + 2 μg GLA-SE and minimal for the remaining groups (Fig. 2b). We observed that the predominant profile for CD4^+^ T cells elicited by ID93 + GLA-SE was a polyfunctional CD154^+^TNF^+^IL-2^+^ phenotype followed by several other polyfunctional, bifunctional, and monofunctional phenotypes containing these markers (Fig. 3a–c). The profiles for the 2 μg ID93 + 2 μg GLA-SE and 10 μg ID93 + 2 μg GLA-SE groups were similar. A greater proportion of CD4^+^ T cells co-expressing two or three cytokines was observed in the adjuvant containing than in the antigen alone groups (Fig. 3d). Specifically, for the adjuvanted vaccine recipients there was a decrease in proportion of single positive TNF^+^ and IFNγ^+^ CD4^+^ T cells and an increase in proportion of TNF^+^IL-2^+^ CD4^+^ T cells. Taken together, the T cell response measurements indicate that the GLA-SE adjuvant enhances both the magnitude and polyfunctionalilty of vaccine-specific CD4^+^ T cells.

### Antibody effector function profiles

**Fig. 4:**
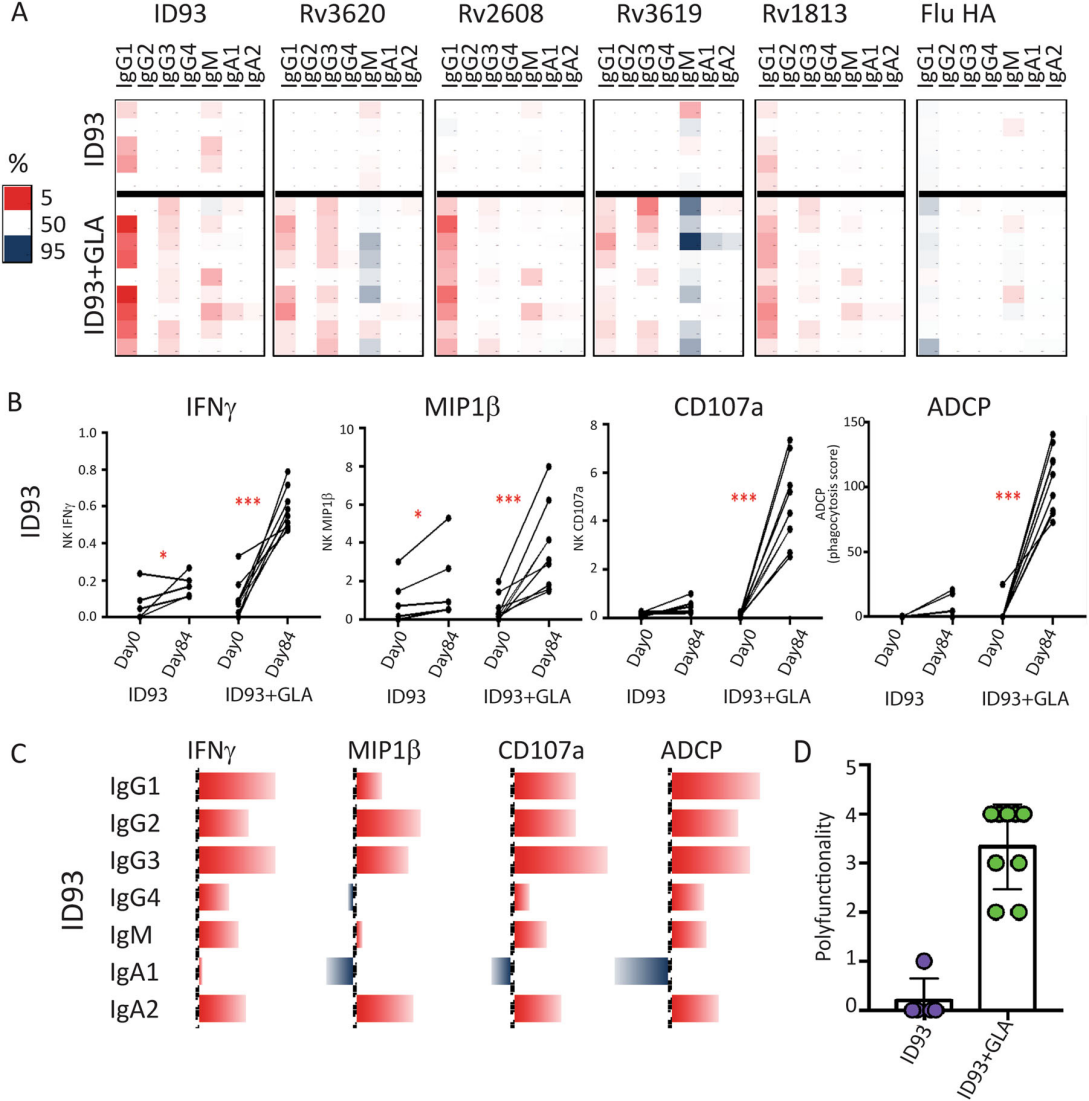
Vaccine induced antibody response profiles. **a** Heatmap shows changes in vaccine antigen-specific and control influenza hemagglutinin-specific antibody isotype titers after vaccination. Each row represents an individual. Each column represents an antibody isotype. The vaccine regimen is specified on the left with legend showing that red represents the top 5th percentile in the amount of change (large changes) and blue represents the 95th percentile in the amount of change (small changes). **b** Changes in ID93-specific antibody-dependent cellular cytotoxicity (ADCC) NK cell production of IFNγ, MIP1β, and CD107a, and antibody-dependent cellular phagocytosis (ADCP) are plotted. Each line represents each individual before (Day 0) and after (Day 84) vaccination and individuals are grouped by vaccine regimen. Statistical significance calculated by Wilcoxon matched pairs signed rank test is indicated (*p < 0.05, ***p < 0.001). **c** Spearman correlation coefficients between ID93 antibody-specific isotypes and ID93-specific antibody effector functions are depicted by bars with red denoting positive and blue denoting negative values. **d** Polyfunctionality as defined by total number of ID93-specific antibody effector functions (NK cell-mediated IFNγ, MIP1β, and CD107a, and antibody-dependent cellular phagocytosis) are graphed on a per individual basis. Each individual is represented by a dot, and individuals are grouped into vaccine regimens as noted (purple = ID93, green = ID93 + GLA-SE)

Across multiple isotypes and subclasses, vaccine-specific antigens were augmented in the setting of GLA-SE adjuvant with minimal effect on control influenza HA antibody titers (Fig. 4a); the highly functional antibody subclasses IgG1 and IgG3^23^ were upregulated. Antibody effector functions were also significantly modulated by the addition of the adjuvant. Specifically, an antibody-dependent cellular cytotoxicity (ADCC) assay^24^ indicated that ID93-specific antibody-mediated NK cell degranulation and activation exhibited enhanced IFNγ, MIP1β, and CD107a upregulation following the addition of the adjuvant compared to the administration of ID93 alone (Fig. 4b). Similarly, antibody-mediated cellular phagocytosis (ADCP)^25^ of ID93 adsorbed beads was not induced by ID93 alone but significantly increased by ID93 + GLA-SE. The various effector functions were not correlated with one specific but rather multiple subclasses and isotypes (Fig. 4c) that likely act in a coordinated manner to recruit antibodies with activities that have been linked to Mtb restriction in vitro.^11^ Individuals who received ID93 + GLA-SE demonstrated more polyfunctional antibody responses able to recruit multiple NK cell functions and increase monocyte phagocytosis compared to those who received ID93 without GLA-SE (Fig. 4d).

## Discussion

We found that the Mtb protein subunit antigen, ID93, was safe in healthy US adults, and elicited robust antibody and CD4^+^ T cell immune responses when combined with GLA-SE.

In this study, ID93 alone and ID93 + GLA-SE were well tolerated and the most frequent AEs were injection site pain, headache, and fatigue. Injection site pain was more frequently observed in the groups of subjects that received ID93 + GLA-SE (83–100%) as compared to ID93 alone (17 and 33%). These data agree with other studies administering GLA-SE in combination with a wide variety of antigens for other infectious diseases.^26–28^ The addition of adjuvant has previously been found to increase the rate and severity of local and systemic reactogenicity compared to the antigen alone. This is similar to clinical experience with another TLR4 agonist (MPL®; GSK Biologicals) and is to be expected of a potent immunomodulator.^29,30^

Induction of a polyfunctional T cell response is associated with protection against Mtb in a number of preclinical challenge animal models.^17,31–33^ We observed substantial CD4^+^ responses in all ID93 + GLA-SE regimens. A linear dose–response relationship was not observed and differences in CD4^+^ T cell responses in the whole blood ICS assay (Fig. 3b) were not statistically significant. The antigen Rv2608 was immunodominant, but CD4^+^ responses were also detected against Rv1813, Rv3619, and Rv3620 in this naive, non-BCG vaccinated and QuantiFERON-negative population. CD8^+^ T cell-mediated responses were minimal, consistent with results from other adjuvant subunit vaccines designed to elicit Th1 responses.^34,35^ Inclusion of the GLA-SE adjuvant led to higher T cell response magnitudes than antigen alone. Furthermore, the cytokine profiling results indicated that inclusion of GLA-SE also improved the overall T cell response quality by eliciting a significantly higher proportion of polyfunctional CD4^+^ T cells including TNF^+^ IL-2^+^ IFNγ^−^ cells, similar to immune responses observed in mice immunized with ID93 + GLA-SE.^17,19,20,22,36^ In preclinical challenge studies a stronger Th1 rather than a Th2 response is needed to limit mycobacterial growth and is associated with reduced immunopathology.^19,37^ Although memory markers were not assessed here, the T cell cytokine profiles are associated with a less differentiated central memory phenotype, which has been found capable of entering the lung tissue and providing protection from TB in mice.^38,39^

Vaccination with ID93 + GLA-SE induced a significantly higher antibody response than ID93 alone, which peaked after two injections in 100% of recipients and did not differ between varying antigen and adjuvant doses. Preclinical studies suggest that Mtb antigen-specific antibody responses have a protective role, perhaps by reducing extra-pulmonary dissemination or by Ab-mediated enhancement of phagocytosis.^11,12,40–42^ Adjuvant-driven IgG responses targeted all four antigens and were comprised predominantly of IgG1 and IgG3 subclasses, which are associated with several beneficial antibody Fc-mediated effector functions. Only antibodies from ID93 + GLA-SE recipients mediated NK cell degranulation/activation and THP1 monocyte mediated antibody-dependent phagocytosis, showing differential Fc receptor engagement in ADCC and phagocytosis, respectively, with potential implications for all Fc receptor bearing immune cells including macrophages and neutrophils.^43^ Moreover, antibody functions were not correlated with one but rather multiple subclasses and isotypes, similar to those associated with Mtb restriction in vitro.^11^ To our knowledge, no other candidate TB vaccine has been evaluated for such antibody effector functions. These findings suggest that the inclusion of GLA-SE as an adjuvant in rational vaccine design augments antibody effector functional profiles.

Several factors limited the conclusions that can be drawn from these studies. These include the small group sample sizes and restricted analysis of certain time points and subject groups for some analyses. Larger group sizes might have enabled statistical significance to be achieved for CD4 T cell response magnitudes in between the different groups. T cell responses were only analyzed through Day 70, and thus we cannot comment on the durability of vaccine-induced CD4 T cell responses. Humoral responses were evaluated and persisted above baseline 6 months after the last injection. CD4 T cell cytokine profiles were assessed in ID93-stimulated whole blood only for Cohorts 1 and 2 due to cost constraints and therefore whether 2 μg or 5 μg GLA-SE differed in this regard remains unknown. Similarly, antibody functional profiling was only performed for Cohorts 1 and 2 subjects.

This first-in-human trial was conducted in a healthy, BCG-naive and QuantiFERON-negative, non-endemic population. One might expect the ID93 vaccine, which contains antigens present in both BCG and Mtb, to induce a higher and/or different cellular and humoral response among people who are “primed” with BCG or with LTBI (QuantiFERON-positive), as has been observed previously.^35,44–46^ Therefore, subsequent clinical studies have been recently conducted, including a phase 1b trial in QuantiFERON-negative and QuantiFERON-positive individuals (TBVPX-114; NCT01927159) and a phase 2a trial administering the vaccine at the end of TB treatment (TBVPX-203; NCT02465216). Future studies will evaluate the ability of the vaccine candidate to prevent infection among BCG vaccinated individuals in TB-endemic areas or to shorten the duration of therapy or reduce relapse among persons completing chemotherapy for TB. In conclusion, the safety and immunogenicity profiles reported for varying regimens of the ID93 + GLA-SE candidate vaccine support further clinical development of a dose regimen of 2 μg ID93 + 2μg GLA-SE.

## Materials and methods

### Vaccine

ID93 is a recombinant fusion protein comprised of four Mtb antigens: Rv1813 (hypothetical protein expressed in in vitro latency models^47,48^); Rv2608 (surface protein of the PE/PPE family^49^); and Rv3619 and Rv3620 (secreted proteins belonging to the same ESX family as ESAT-6 and CFP-10^50^). The fusion protein is combined with the adjuvant GLA-SE. ID93 was manufactured under cGMP by University of Iowa—Center for Biocatalysis and Bioprocessing (Coralville, IA) and fill/finished by University of Iowa—Pharmaceuticals (Iowa City, IA). GLA was manufactured under cGMP by Avanti Polar Lipids (Alabaster, AL) and GLA-SE was formulated, filled, and finished by IDRI (Seattle, WA).

### Study design

TBVPX-113 was a phase 1, randomized, double-blinded, dose-escalation evaluation of the safety, tolerability, and immunogenicity of two dose levels of the ID93 antigen administered intramuscularly (IM) either alone or in combination with one of two dose levels of the GLA-SE adjuvant (ClinicalTrials.gov identifier: NCT01599897). The study was conducted in 60 HIV-negative, healthy US adults who were BCG-naive and negative for prior exposure to Mtb (QuantiFERON-negative) at enrollment. The sample size for this study was selected as adequate to detect frequent AEs. This research was conducted in accordance with all relevant guidelines and procedures under an IND with the U.S. FDA, and was approved by the MidLands Independent Review Board (Overland Park, KS, USA).

**Fig. 5:**
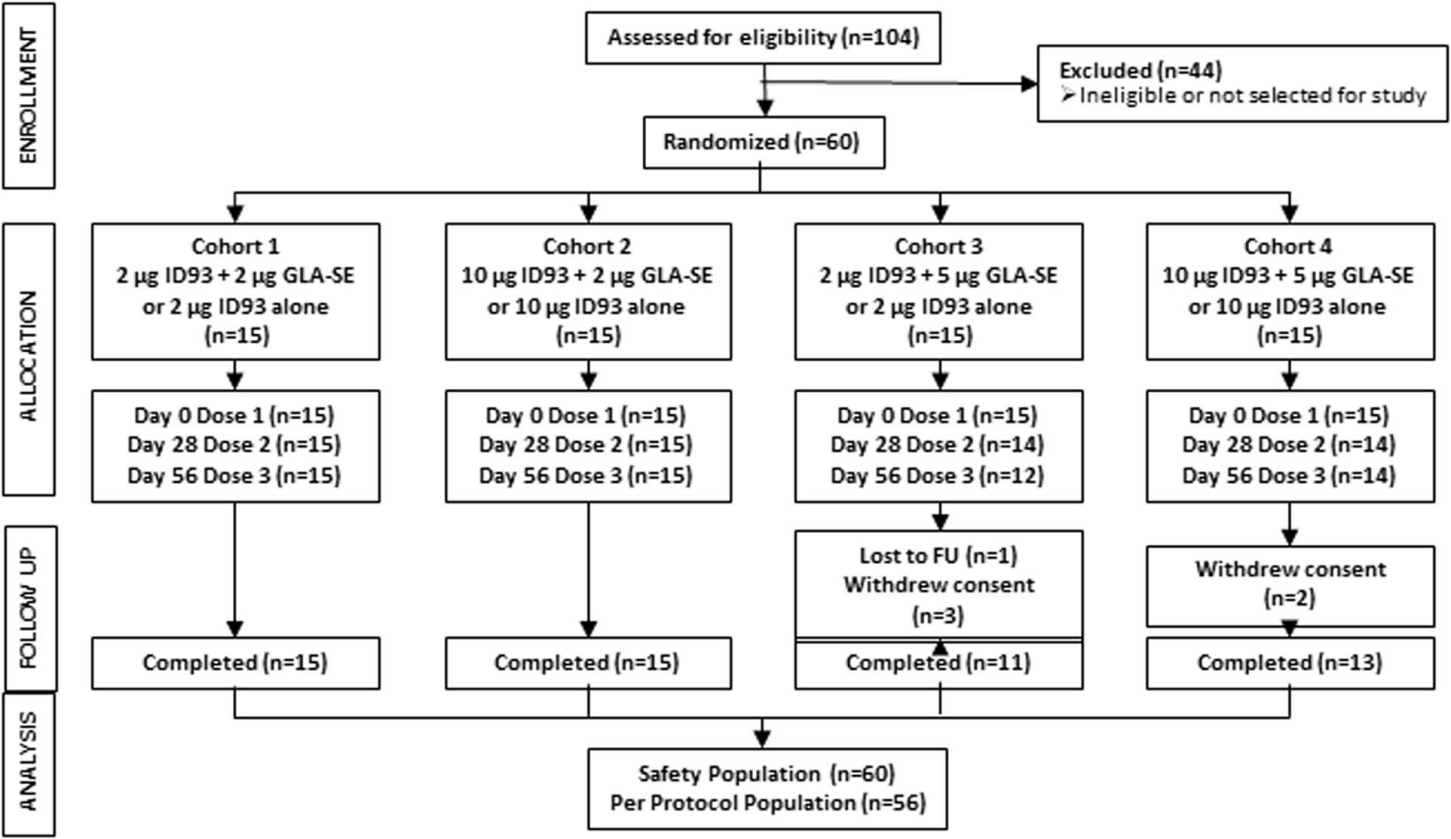
Clinical trial CONSORT diagram for TBVPX-113. Flow chart shows the number of subjects entering the study from enrollment, allocation, and follow-up (FU). Subjects missing the second and third injection dose either withdrew consent or were lost to FU. Subjects were in FU for 1 year after the third injection. The Safety Population is defined as all subjects who received at least one study injection; the Per Protocol Population comprises subjects who received all three study injections and completed Day 84. All subjects received at least one study injection and were included in the analyses of safety and immunology

Subjects were consented in writing, screened, enrolled, and randomized in a 4:1 ratio such that 12 subjects received ID93 + GLA-SE and 3 subjects received ID93 in four sequential cohorts as shown in Fig. 5. Cohort 1: 2 μg ID93 + 2 μg GLA-SE or 2 µg ID93 alone; Cohort 2: 10 μg ID93 + 2 μg GLA-SE or 10 µg ID93 alone; Cohort 3: 2 μg ID93 + 5 μg GLA-SE or 2 µg ID93 alone; Cohort 4: 10 μg ID93 + 5 μg GLA-SE or 10 µg ID93 alone. Subjects received a total of three IM study injections at Days 0, 28, and 56. Safety assessments were performed 1, 3, 7, 14, and 28 days after each study injection. Solicited and unsolicited AEs were collected for 28 days after each study injection, including clinical laboratory assessments (7 days after each injection) and vital signs (30 and 60 min after each injection). Serious adverse events (SAEs) and AEs of special interest (defined as potentially immune-mediated events) were collected for the duration of the study. Subjects were followed for 1 year after the third study injection administration (to Day 420). More information is provided in the Supplemental Materials.

### Primary immunogenicity evaluation

Vaccine-specific antibody responses were measured by enzyme-linked immunosorbent assay (ELISA). Endpoint titer for total IgG specific for ID93 fusion protein and its components were evaluated (RV1813, RV2608, RV3619, and Rv3620). Sera collected at Days 0, 28, 56, 84, and 238 from all cohorts was evaluated. Details are described in the Supplemental Materials.

Antigen-specific T cell responses were evaluated in cryopreserved PBMCs using a 6-h stimulation with DMSO or peptide pools comprising Rv1813, Rv2608, Rv3619, or Rv3620 followed by intracellular cytokine staining (ICS) and flow cytometry.^51^ Frequencies of CD4^+^ and CD8^+^ T cells positive for any antigen-specific marker (IFNγ, TNF, IL‐2, CD154, IL-4/IL‐22, and/or IL‐17) were quantitated. Unstimulated (DMSO) values were subtracted. Details are provided in the Supplemental Materials.

T cell responses were also evaluated in fresh whole blood using a 12-h stimulation with ID93 fusion protein or DMSO.^51,52^ Frequencies of CD4^+^ and CD8^+^ T cells positive for any antigen-specific marker (IFNγ, TNF, IL‐2, CD154, and/or IL‐2) were quantitated. Unstimulated (DMSO) values were subtracted. Details are provided in the Supplemental Materials.

### Antigen-specific IgG subclass quantitation

A customized Luminex subclass assay was used to quantify the relative concentration of each antigen-specific antibody isotype and subclass at selected time points.^53^ Carboxylated microspheres (Luminex) were coupled with ID93 protein antigen by covalent NHS-ester linkages via EDC and NHS (Thermo Scientific) per the manufacturer’s instructions. Antigen-coated microspheres (3750 per well) were added to a 96-well glass bottom plate (Grenier). Each serum sample (Days 0 and 84, all cohorts) at two different dilutions (1:100 and 1:30) was added to duplicate wells of a 96-well plate and incubated for 16 h at 4 °C. The microspheres were washed, and IgG1-specific, IgG2-specific, IgG3-specific, IgG4-specific, bulk IgG, IgA1-specific, IgA2-specific, or IgM-specific detection reagents (Southern Biotech) were added for 2 h at room temperature. The beads were then washed and read on a Bio-Plex 200 System. The background signal, defined as MFI of microspheres incubated with PBS, was subtracted.

### THP1 phagocytosis assay

The THP1 phagocytosis assay of antigen-coated beads was conducted as previously described using serum collected at Days 0 and 84, all cohorts.^25^ Details are provided in the Supplemental Materials. Phagocytic scores are presented as the integrated MFI (% bead-positive frequency × MFI/10,000).^54^

### Antibody-dependent NK cell activation

ELISA-based Ab-dependent NK cell activation assay was modified for use with ID93 antigen.^24^ Briefly, ELISA plates (Thermo Fisher NUNC MaxiSorp flat bottom) were coated with ID93 (3 ng/well) or BSA as a negative control at 4 °C for 16 h. Serum samples from Days 0 and 84 from all cohorts were diluted 1:100 and added to each well. NK cells were isolated from whole blood from HIV-seronegative donors with RosetteSep (STEMCELL Technologies) and cultured overnight with IL-15 (1 ng/mL). NK cells (5 × 10^4^ per well), anti-CD107a-phycoerythrin (PE)-Cy5 (catalog number 555801, BD), brefeldin A (10 mg/mL Sigma), and GolgiStop (BD) were added to each well, and the plates were incubated for 5 h at 37 °C. Cells were then stained for surface markers using anti-CD16–allophycocyanin (APC)-Cy7 (catalog number 557758, BD), anti-CD56-PE-Cy7 (catalog number 557747, BD), and anti-CD3-AlexaFluor 700 (catalog number 557943, BD), and then stained intracellularly with anti-IFNγ-APC (catalog number 554702, BD) and anti-MIP1β-PE (catalog number 550078, BD) using Fix and Perm A and B solutions (Thermo Fisher Scientific). Fixed cells were analyzed by flow cytometry. NK cells were defined as CD3− and CD16/56+.

### Statistical analysis

Safety and immunogenicity analyses were performed on data obtained from subjects who received at least one study injection. Data were transformed as appropriate prior to analysis and included assessment of responses at all pre-injection and post-injection time points, as well as change from pre-injection to each post-injection time point, by dose cohort and treatment regimen. More information is provided in the Supplemental Materials.

### Data availability

The underlying data reported in this paper are available from the corresponding author upon reasonable request.

## Electronic supplementary material

Supplemental Materials

## Electronic supplementary material

**Supplementary information** accompanies the paper on the *npj Vaccines* website (10.1038/s41541-018-0057-5).
