# Supplementary material for: The TLR-4 agonist adjuvant, GLA-SE, improves magnitude and quality of immune responses elicited by the ID93 tuberculosis vaccine: first-in-human trial
Source: NPJ Vaccines. 2018 Sep 4;3:34. doi: 10.1038/s41541-018-0057-5 (PMC6123489; doi:10.1038/s41541-018-0057-5)
Supplement: Supplementary file 1 — Supplemental Materials [file 41541_2018_57_MOESM1_ESM.doc]

## Supplemental Materials

**Supplemental Methods**

**Study Design**

Subjects and site personnel involved in observing the subjects were blinded as to treatment regimen. The trial was conducted at Johnson County Clin-Trials (Lenexa, Kansas). Cohorts were enrolled sequentially and blinded safety data were reviewed before allowing dose-escalation and enrollment of each subsequent cohort. This study was run under an IND with the U.S. FDA and was approved by Midlands IRB (Overland Park, KS).

Subjects were sequentially assigned to a Dose Cohort based on time of completion of screening and then randomized within each Dose Cohort to a treatment regimen based on a sequential series of two-digit randomization numbers linked to a randomly-generated sequence of treatment assignments (randomization schedule).

The occurrence of local injection site reactions was assessed at 30 minutes, and 1, 3, 7, 14, and 28 days following each study injection. The occurrence of the specific solicited local reactions (pain, redness, and swelling at the site of injection) and systemic symptoms (fever, myalgia, arthralgia, fatigue, headache, anorexia, hives, and chills) was evaluated through 28 days after each injection (with diary cards completed by subjects for 7 days following each study injection). Blood samples were collected for measurement of hematology and serum chemistry parameters at screening and on Days 7, 35, and 63.

The primary safety endpoints were the proportion of subjects with unsolicited AEs, serious AEs, AEs of special interest, local injection site reactions, and specific solicited systemic reactions to the study injections from Days 0 to 84. The investigator assessed their relation to study injections, and severity based on the Food and Drug Administration Guidance for Industry Toxicity Grading Scale for Healthy Adults Enrolled in Preventive Vaccine Clinical Trials, 2007.

**Specimens Analyzed**

Specimens from all study subjects were included in the primary immunogenicity assessments, ELISA and PBMC ICS. For ELISA, all serum collection time points were evaluated. For PBMC ICS, baseline and 2 weeks post each vaccination (Days 0, 14, 42, and 70) were evaluated. Specimens from only Cohort 1 and 2 subjects were used for exploratory analyses, due to cost constraints, including whole blood ICS, antibody subclass analysis, phagocytosis assay, and NK cell activation.

**Enzyme-Linked Immunosorbent Assays (ELISAs)**

Antibody responses were measured by enzyme-linked immunosorbent assay (ELISA). Serum collected at Days 0, 28, 56, 84, and 238 from all cohorts were evaluated. Endpoint titers for total IgG was evaluated for ID93 fusion protein and each component protein antigen. IgG subclasses (IgG1, IgG2, IgG3, and IgG4) were evaluated for ID93 fusion protein.

384-well High Binding Plates (Corning, NY) were coated with 50 μL ID93 or ID93 components at 1 μg/mL in coating buffer (eBiosciences, CA) overnight (ON) at 4^o^C. Wells were washed 5X with wash buffer (PBS/0.1% Tween-20) followed by blocking with 1% BSA/ PBS/0.05% Tween ON at 4^o^C. Samples were serially diluted with diluent (0.1% BSA/PBS/0.05% Tween) to create a 10x master plate, starting at 1:10 and stepping by 4 fold dilutions for 12 points of a curve. 5 μl was transferred from the master plate to 5 assay plates containing 45 μl diluent to achieve a top curve point of a 1:100 dilution and incubated overnight at 4^o^C. Plates were washed 5 times, followed by the addition of anti-human IgG1, IgG2, IgG3, IgG4, or Protein G peroxidase-labeled in diluent according to manufacturer’s recommendation (Life Technologies, CA) and incubated for 1 hour at room temperature. Plates were washed 5 times and developed with 50 μL TMB SureBlue Peroxidase Substrate (KPL, MD) followed by adding 25 μL 1N H_2_SO_4_. Optical density (OD) was read at 450nm and 570nm. Endpoint titers were calculated using OD 450nm minus 570nm for interpolating unknowns from the last value greater than the threshold given by normal human plasma pool using a 4-parameter logistic model (Parameter 208) XL-Fit software as a Microsoft Excel add-in.

**Intracellular Cytokine Staining (ICS) Assays**

**PBMC ICS -** PBMC collected at Days 0, 14, 42, and 70 from all cohorts were evaluated. Briefly, PBMC were thawed and rested overnight. Cells (1x10^6^ cells/condition) were stimulated with Rv1813, Rv2608, Rv3619, or Rv3620 peptide pools (JPT Peptide Technologies, Germany) in round-bottom 96-well plates. Each antigen pool consisted of 15mer peptides overlapping by 11 amino acids spanning the entire length of the antigen, dissolved in DMSO (Sigma, St. Louis, MO, USA), and used at a final concentration of 1µg/peptide/mL. Peptides were diluted in R10 medium containing CD107a-Alexa488 (clone H4A3; catalog number 328610, BioLegend, San Diego, CA, USA), GolgiStop and GolgiPlug (BD Biosciences, San Jose, CA, USA). DMSO (Sigma) and SEB (Sigma, 0.5 µg/mL) were used as negative positive controls, respectively. Following 6-7 hours of stimulation at 37°C and 5% CO2, cells were washed and stained using LIVE/DEAD aqua viability dye (Life Technologies, catalog number L34966, Waltham, MA, USA). Cells were then washed and stained with anti-CCR7-Brilliant Violet 605 (clone G043H7; catalog number 353224, BioLegend). For cohorts 1 and 2, Days 0, 14, 42, and 70, cells were then washed and stained using anti-CD4-APC-eFluor 780 (clone RPA-T4; eBioscience, catalog number 47-0049-41, Waltham, MA, USA), anti-CD8-Alexa Fluor 700 (clone HIT8a; catalog number 300920, BioLegend), anti-CD45RO-Brilliant Violet 785 (clone UCHL1; catalog number 304234, BioLegend), anti-CD14-V500 (clone M5E2; catalog number 561391, BD Biosciences), and anti-CD19-V500 (clone HIB19; catalog number BDB555412, BD Biosciences). Cells were then washed, fixed and permeabilized with BD Cytofix/Cytoperm (BD Biosciences) and then stained intracellularly using anti-CD3-ECD (clone UCHT1; catalog number IM2705U, Beckman Coulter, Indianapolis, IN, USA), anti-IFN-γ-Brilliant Violet 421 (clone 4S.B3; catalog number 502532, BioLegend), anti-TNF-PE-Cy7 (clone Mab11; catalog number 554514, BD Biosciences), anti-CD154-PE-Cy5 (clone TRAP1; catalog number 555701, BD Biosciences), anti-IL-2-PE (clone MQ1-17H12; catalog number BDB554566, BD Biosciences), and anti-IL-4-APC (clone MP4-25D2; catalog number 554486, BD Biosciences). For corhorts 3 and 4, and all Day 238 samples, anti-IL-4-APC was replaced with anti-IL-22-APC (clone IL22JOP; catalog number 17-7222-82, eBiosciences) since no vaccine-related staining had been observed with the IL-4 antibody and the anti-IFN-γ was replaced with a different clone that resulted in reduced background noise (clone B27; catalog number 562988, BD Biosciences). For cohort 3, Days 0, 14, 42, and 70, CD8 was stained intracellularly to compensate for down-regulation of CD8 on the cell surface. Cells were then washed, fixed, and acquired on a BD LSR II flow cytometer and then analyzed using FlowJo software (TreeStar Inc, USA).

**Whole blood ICS -** T cell responses were also assessed in antigen-stimulated fresh whole blood specimens. For the antigen-stimulation, 900 µL of whole blood was added to 100 µL of stimulant: DMSO (negative control), PHA (5 μg/mL, positive control, Fisher) or ID93 protein (10 μg/mL). Blood was incubated for 12 hours at 37°C and 5% CO_2_ with anti-CD28 (eBioscience), anti-CD49d (eBioscience) and anti-CD107a-PE-Cy7 (clone H4A3; catalog number 561348, BD Biosciences) antibodies. Brefeldin A (eBiosciences) was added for the last 5 hours. Cells were then lysed and cryopreserved. Intracellular cytokine staining was performed on specimens collected at Days 0, 42, and 70 from Cohorts 1 and 2. Cells were thawed and stained with: anti-CD3-BV421 (clone UCHT1; catalog number 562426, BD Biosciences), anti-CD4-BV786 (clone SK3; catalog number 563877, BD Biosciences) and anti-CD8-PerCP-Cy5.5 (clone SK1, catalog number 341051, BD Biosciences). Cells were fixed and permeabilized with BD Cytofix/Cytoperm (BD Biosciences) then stained with intracellular markers: anti-IFN-γ-AF700 (clone B27; catalog number 557995, BD Biosciences), anti-TNF-α-PE-Cy-7 (clone Mab11; catalog number 25-7349-82, eBiosciences), anti-IL-2-FITC (clone 5344.111; catalog number 340448, BD Biosciences) and anti-CD154-PE-Cy5 (clone 24-31; catalog number 310808, Biolegend). Cells were washed, fixed with paraformaldehyde (EM Sciences) and collected on a BD Fortessa. Analysis was performed in Flowjo (TreeStar Inc).

### **THP1 Phagocytosis Assay**

The THP1 phagocytosis assay of antigen-coated beads was conducted as previously described using serum collected at Days 0 and 84, all cohorts). Briefly, ID93 protein antigen was biotinylated with Sulfo-NHS-LC Biotin (Thermo Fisher Scientific). Biotinylated antigens were incubated with 1 μm fluorescent neutravidin beads (Invitrogen) at 4°C for 16 hours. Excess antigen was washed away. Antigen coated beads were incubated with serum samples diluted 1:100 for 2 hours at 37°C. Excess serum was washed away. THP1 cells (5 x 10^4^ per well) were added and incubated at 37°C for 16 hours. Bead uptake was measured in fixed cells using flow cytometry on a BD LSRII equipped with high-throughput sampler. Phagocytic scores are presented as the integrated MFI (% bead-positive frequency x MFI/10,000).

### **Statistical Analysis**

Safety and immunogenicity analyses were performed on data obtained from subjects who received at least one study injection. Data were transformed as appropriate prior to analysis and included assessment of responses at all pre- and post-injection time points, as well as change from pre-injection to each post-injection time point, by dose cohort and treatment regimen. A total of 104 individuals were screened for this study and 60 subjects were enrolled and randomized. All 60 subjects received the first study injection on Day 0. Fifty-eight (96.7%) subjects received the second study injection on Day 28, and 56 (93.3%) subjects received the third study injection on Day 56. No imputation for missing data was performed.

The primary variable for evaluation of the safety profile was the number and percentage of unsolicited and solicited AEs recorded at all available post-vaccination time points. For all presentations of AEs, additional summaries based on reporting period of AEs following each study injection were also presented. Additional summaries presented the number (percentage) of subjects with AEs by severity and by relationship to study injection.

For ELISAs, the geometric mean titer and 95% confidence intervals were determined using GraphPad Prism software. Statistical significance between adjuvant containing regimens and protein alone regimens at each time point was evaluated by 2 Way ANOVA and Tukey’s multiple comparisons test (GraphPad Prism).

For PBMC ICS assay, CD4+ and CD8+ T cell responses to the four ID93 peptide pools (Rv1813, Rv2608, Rv3619, Rv3620) at all specified study time points, as measured by the flow cytometry-based ICS assay, were used to describe the immunogenicity of ID93 antigen alone and in combination with GLA-SE adjuvant. Percentage T cell response was summarized by treatment regimen, T cell type (CD4+ and CD8+) and by stimulation antigen using median DMSO-subtracted cytokine (CD107a, CD154, IFN-γ, IL-2, IL-4/IL-22, IL-17A, or TNF, alone or in any combination) response and associated 95% confidence intervals (CI) based on order statistics, and immunology time point.

For whole blood ICS assay data, Graphpad Prism was used to apply the Wilcoxon matched pairs signed rank test to compare frequencies of ID93-specific cytokine response between Day 0 and Day 42 time points.

For antibody functional profiling assays, statistical significance was calculated by Wilcoxon matched-pairs signed rank test. Spearman correlation coefficients between ID93 antibody specific isotypes and ID93 specific antibody effector functions were also determined.
